# An Update on the Entomology, Virology, Pathogenesis, and Epidemiology Status of West Nile and Dengue Viruses in Europe (2018–2023)

**DOI:** 10.3390/tropicalmed9070166

**Published:** 2024-07-20

**Authors:** Federica Frasca, Leonardo Sorrentino, Matteo Fracella, Alessandra D’Auria, Eleonora Coratti, Luca Maddaloni, Ginevra Bugani, Massimo Gentile, Alessandra Pierangeli, Gabriella d’Ettorre, Carolina Scagnolari

**Affiliations:** 1 Laboratory of Virology, Department of Molecular Medicine, Sapienza University of Rome, 00185 Rome, Italy; leonardo.sorrentino@uniroma1.it (L.S.); matteo.fracella@uniroma1.it (M.F.); alessandra.dauria@uniroma1.it (A.D.); coratti.1847223@studenti.uniroma1.it (E.C.); massimo.gentile@uniroma1.it (M.G.); alessandra.pierangeli@uniroma1.it (A.P.); carolina.scagnolari@uniroma1.it (C.S.); 2 Department of Public Health and Infectious Diseases, Sapienza University of Rome, 00185 Rome, Italy; luca.maddaloni@uniroma1.it (L.M.); ginevra.bugani@uniroma1.it (G.B.); gabriella.dettorre@uniroma1.it (G.d.)

**Keywords:** WNV, DENV, mosquito, pathogenesis, epidemiology, Europe

## Abstract

In recent decades, increases in temperature and tropical rainfall have facilitated the spread of mosquito species into temperate zones. Mosquitoes are vectors for many viruses, including West Nile virus (WNV) and dengue virus (DENV), and pose a serious threat to public health. This review covers most of the current knowledge on the mosquito species associated with the transmission of WNV and DENV and their geographical distribution and discusses the main vertebrate hosts involved in the cycles of WNV or DENV. It also describes virological and pathogenic aspects of WNV or DENV infection, including emerging concepts linking WNV and DENV to the reproductive system. Furthermore, it provides an epidemiological analysis of the human cases of WNV and DENV reported in Europe, from 1 January 2018 to 31 December 2023, with a particular focus on Italy. The first autochthonous cases of DENV infection, with the most likely vector being *Aedes albopictus*, have been observed in several European countries in recent years, with a high incidence in Italy in 2023. The lack of treatments and effective vaccines is a serious challenge. Currently, the primary strategy to prevent the spread of WNV and DENV infections in humans remains to limit the spread of mosquitoes.

## 1. Introduction

In the past few decades, the changing climate is thought to have affected ecosystems. This has led to changes in the natural habitats of many animal species [1]. As temperatures rise, the metabolism of insects increases and their life cycle shortens. In this context, the activity of mosquitoes is greater and, consequently, the feeding, due to the effect of the bite, triggers a greater transmission of several pathogens, including those of the *Orthoflavivirus* genus, which cause diseases in humans and other animals, wild or domestic [2]. As a result, among orthoflavivirus infection, the number of human cases of West Nile virus (WNV) infections has increased not only in the United States of America (USA) [3] but also in Europe, particularly Central and Mediterranean Europe [4,5]. Data from the Centers for Disease Control and Prevention’s (CDC) National Arbovirus Surveillance System (ArbonET) collected for 2023 showed 2566 US WNV cases [5], more than double the number reported in 2022 [6]. In parallel, the European Centre for Disease Prevention and Control (ECDC) has reported nearly 700 human cases, with the highest frequency of WNV infections in Italy (i.e., 336 human cases of WNV infections) [7].

Population growth, urbanization, and travel, as well as resistance to insecticides, whether in the form of larvicides or adulticides, have been the main cause of dengue virus (DENV) human infection worldwide over the last 50 years [8]. In 2023, more than 6 million cases and more than 6000 dengue-related deaths were reported in 92 countries worldwide [9]. Since the beginning of 2024, more than 10 million DENV cases and more than 5000 dengue-related deaths have been reported globally [10]. In particular, Brazil is a country with approximately 3 million laboratory-confirmed cases of dengue according to the World Health Organization (WHO) [11]. In general, DENV is recognized as endemic in tropical and sub-tropical countries, with the majority of cases reported in Brazil, Paraguay, Argentina, and Bolivia [9]. However, an increasing number of autochthonous/non-travel-associated DENV cases have been described in several European countries in recent years. In particular, Italy reported the highest number of confirmed cases (n = 82) in 2023 [9]. As far as 2024 was concerned, a total of 283 DENV cases have been reported in Italy, all associated with travel abroad [12]. No deaths have been reported [12].

Albeit in a small percentage of infections, WNV and DENV can be the cause of severe disease such as West Nile neuroinvasive disease (WNND) (i.e., meningitis, encephalitis, and/or acute flaccid paralysis) and severe dengue illnesses [i.e., dengue hemorrhagic fever (DHF) and dengue shock syndrome (DSS)] [13,14]. The other clinical illnesses caused by WNV and DENV [such as those related to WNV fever and dengue fever (DF)] are equally significant public health concerns due to their impact on human health, the direct costs of treatment, and the indirect costs of sick leave. In addition to the clinical manifestations associated with DENV infection, other viruses and their associated diseases are transmitted by DENV vectors, such as Zika and Chikungunya [15].

To date, specific antiviral treatments for human use against WNV and DENV infections have not been approved [16]. Several human clinical studies have been conducted with vaccines against WNV [17]. However, the live attenuated vaccine ChimeriVax-WN02 is the only candidate against WNV due to its high immunogenicity, safety, and tolerability observed in all-age individuals [18,19]. However, vaccine efficacy and cost effectiveness are significant concerns that limit WNV vaccination programs. The only licensed vaccines to prevent DENV infections are CYD-TDV (known as Dengvaxia) and TAK-003 (known as QDenga). Specifically, the first was approved in Europe in 2019 and can be administered to individuals aged between 6 and 45 years with a history of DENV infection. TAK-003, licensed in Europe in 2022, is approved for individuals aged 4 years and older, regardless of any previous exposure to DENV. However, vaccine efficacy was demonstrated against DENV-1 and DENV-2, but not against DENV-3, while the low incidence of DENV-4 limits the evaluation against this serotype [20,21].

This review aims to summarize the entomological literature on the mosquito vectors of WNV and DENV and to assess the role of animal hosts in their transmission. Furthermore, this review aims to provide new knowledge on the virological and pathogenic aspects of DENV and WNV infections. Finally, the epidemiology of the circulation of WNV and DENV in Europe from 1 January 2018 to 31 December 2023 is described, with a particular focus on the spread of these viruses in Italy.

## 2. West Nile Virus and Dengue Virus: Insights into the Vector Competence, Host Range, Transmission, Classification, Genome Organization, Virus Genetics, and Pathogenesis

### 2.1. Mosquito Vectors of West Nile Virus

Competent vectors can transmit WNV. Mosquito anatomical barriers regulating midgut infection, midgut escape, salivary gland infection, and transmission influence WNV competence [22,23]. Temperature, an extrinsic factor, significantly influences WNV vector competence. Higher temperatures enhance virus replication in mosquitoes, speeding up dissemination and shortening incubation periods [24]. The main mosquito genus involved in the transmission of WNV is *Culex. Culex pipiens* (Linneaus, 1823) mosquitoes are the primary vectors of WNV due to their seasonal abundance, vector competence, and high infection rates [25]. They serve as amplifying vectors in the bird-to-bird enzootic cycle, as bridging vectors in the bird-to-mammalian epizootic cycle, and as reservoirs [26]. The vector competence of *Cx. pipiens* is similar in northern and southern Europe. Its transmission rate increases between 18 °C and 28 °C, suggesting a temperature-dependent vector competence [26]. In addition to *Cx. pipiens* and *Culex Quinquefasciatus* (Say, 1823), other mosquito vectors of WNV are *Culex univittatus* (Theobald, 1901), *Culex theileri* (Theobald, 1903), *Culex perexiguus* (Theobald, 1903), *Culex modestus* (Ficaldi, 1890), and *Culex neavei* (Theobald, 1906). Climate change is expected to spread WNV to new areas by creating favorable conditions for mosquito vectors. Extreme weather conditions such as droughts, heat waves, and floods affect the life cycle and virus-carrying capacity of mosquitoes, increasing WNV replication and transmission to humans [27]. Table 1 shows the distribution of the main *Cx*. mosquitoes involved in the transmission of WNV.

### 2.2. Animal Hosts and Secondary Modes of Transmission of West Nile Virus

**Figure 1:**
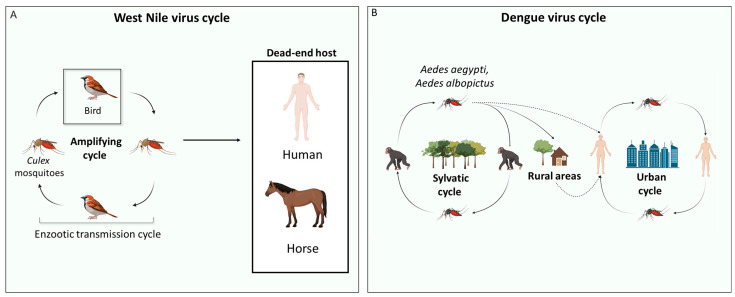
**Life cycles of West Nile virus and dengue virus.** (**A**) The life cycle of West Nile virus (WNV) is shown. The amplifying host is represented by a bird in which, after viral replication, the viral load in the blood is high enough to infect *Culex* mosquitoes. Humans and horses are considered the main hosts of WNV. They are known as dead-end hosts because their blood viremia is not high enough to infect other mosquitoes. (**B**) The sylvatic and urban cycles of dengue virus (DENV) are shown. In the sylvatic cycle, DENV is maintained mainly by *Aedes* mosquitoes and monkeys. The transition from the sylvatic to the urban cycle occurs in two ways: (i) DENV-infected mosquitoes move directly from the sylvatic to the urban area and infect humans; (ii) DENV-infected mosquitoes bite humans living in rural areas, who then move to urban areas. DENV is maintained in the urban cycle by *Ae. aegypti* mosquitoes and humans. The above image was created with https://www.biorender.com/ (accessed on 15 July 2024).

WNV is maintained in an enzootic transmission cycle, with mosquitoes as vectors and birds acting as amplifying hosts. Birds are considered amplifying hosts for WNV because of the high levels of viremia found in their blood [28]. However, WNV can infect incidental hosts, such as humans and horses [29], which are generally considered dead-end hosts because viral replication does not produce significant viremia to allow transmission to feeding mosquitoes (Figure 1A) [28,29,30]. Moreover, WNV can sporadically infect other animal species, such as dogs, cats [31], wild boars [32], sheep [33], alpacas, llamas, and wolves [30]. Similarly to humans and horses, in these dead-end hosts, the viremia generated is low and not sufficient to initiate a new cycle of transmission [30]. Alligators have been described as susceptible to WNV infection and able to develop a viremia of sufficient magnitude to predict at least low competence to infect feeding mosquitoes [34,35]. In addition to the primary mode of transmission of WNV to hosts via competent mosquito vectors, WNV can also be acquired through blood transfusion [36], organ transplantation [37], perinatally, and breastfeeding [38]. WNV, like other orthoflaviviruses, can also be maintained within the mosquito population by vertical transmission from an infected female mosquito to her offspring, even if the rate of this transmission mode is low [39,40,41,42].

### 2.3. Mosquito Vectors of Dengue Virus

*Aedes aegypti* (Linnaeus, 1762) is the main vector for DENV infection [43]. However, even with low efficiency, *Aedes albopictus* (Skuse, 1895) can become infected and transmit DENV [44]. While humans are a dead-end host for WNV infection, as previously shown, they are considered to be an amplifying host for DENV infection (Figure 1B) [45].

The distribution of *Ae. aegypti* and *Ae. Albopictus* is shown in Table 1. At the beginning of the 20th century, *Ae. aegypti* was recorded throughout the Mediterranean basin [46,47], with high abundance in France [48], Greece [49], Italy [50], Russia [51], Portugal [52], and Spain [53]. Although the mosquito was eradicated from the Mediterranean region, it survived along the Black Sea coast [54,55,56]. In addition, *Ae. aegypti*, has been established also in Cyprus since 2022 and could continue to spread to other European countries in the near future [57]. *Ae. albopictus* may be responsible for recent autochthonous cases of DENV in Europe [58]. This phenomenon can be explained by suitable climatic conditions (i.e., higher temperatures and increased rainfall) for the survival of the vector, for the development of the virus in the vector, and for an adequate pool of viremic individuals. In this context, DENV has been detected in *Ae. albopictus*, and the adaptation and spread of this mosquito species in temperate regions could be the cause of recently reported autochthonous DENV cases in Europe [58].

### 2.4. Animal Hosts and Secondary Modes of Transmission of Dengue Virus

DENV is maintained by a sylvatic cycle involving non-human primates [59] (Figure 1B). In rural areas, the spread of DENV to humans can occur when human populations come into contact with the sylvatic cycle [60]. The RNA of the DENV genome has been found in various non-human primates [61,62], i.e., birds [63], bats [62], rodents [64], dogs [65], pigs [66], cattle [67], horses [68], and marsupials [69]. Although DENV is maintained primarily by cross-species transmission between mosquito vectors and vertebrate hosts, vertical transmission of this virus within the mosquito vector population has been proposed as a maintenance mechanism [70], supporting the persistence of DENV in the absence of a recognized host or under unfavorable conditions for mosquito activity [70]. In particular, DENV is transmitted vertically by transovarial transmission, where the virus infects the gonadal tissues of female mosquitoes, and by trans-egg transmission, where the virus infects the eggs during oviposition [71,72]. DENV has a significantly higher effective vertical transmission rate in *Ae. albopictus* than in *Ae. aegypti* mosquitoes [70]. Specifically, DENV-1, one of the four DENV serotypes (i.e., DENV-1, DENV-2, DENV-3, and DENV-4) distinguished by genome sequence variation is better able to transmit vertically than the other DENV serotypes, but the explanation for this serotype-specific phenomenon is still unknown [70].

**Table 1:** Mosquito vector species for West Nile virus and dengue virus and their geographical distribution.

Virus	Vectors Species	Vector Distribution	References
WNV	*Culex pipiens*	Europe, Australia, Asia, Africa, North and South America	[73]
	*Culex quinquefasciatus*	Tropical and sub-tropical regions of Africa, Madagascar, South Asia, North Australia, Mexico, USA, Tropical and sub-tropical South America	[74]
	*Culex univittatus*	Egypt	[75]
	*Culex theileri*	Turkey, Portugal, Spain and Iran	[76]
	*Culex modestus*	Europe, Asia and North Africa	[76]
	*Culex neavei*	Tropical regions of Africa	[76]
	*Culex perexiguus*	South and East Europe, North, East and West Africa, South and West Asia	[76]
DENV	*Aedes aegypti*	Africa, Northeast America, Middle East, Southeast Asia, the Pacific and Indian Islands North Australia, and the Mediterranean Basin *	[77,78,79]
	*Aedes albopictus*	Europe, Middle East, South and East Asia, North, Central and South America, Africa	[77,78,80,81]

The main mosquito vector species for West Nile virus (WNV) and dengue virus (DENV) infections are shown, together with their geographical distributions. * Currently, *Ae. aegypti* is present on the Black See Coast [56] and since 2022 has been established in Cyprus, indicating a potential future spread of this mosquito across Europe [57].

### 2.5. Classification

The *Flaviviridae* family consists of enveloped viruses characterized by a positive single-stranded (ss)RNA genome and an icosahedral nucleocapsid. Four genera have been identified within this family: *Orthoflavivirus*, *Pestivirus*, *Hepacivirus*, and *Pegivirus*. *The Flavivirus* genus, known for its many arthropod-borne viruses, was renamed orthoflavivirus by the International Committee on Taxonomy of Viruses (ICTV) in 2023 to distinguish it from other members of the *Flaviviridae* family [82].

Orthoflavivirus comprises more than 70 viruses, most of which are vector-borne pathogens, and are grouped into serocomplexes based on serological characteristics, including DENV, WNV, Japanese encephalitis virus (JEV), yellow fever virus (YFV), Zika virus (ZIKV), and USUTU virus (USUV) [83].

USUV is considered an emerging orthoflavivirus and its amplifying host is the bird. A meta-analysis study conducted in Modena (northeast Italy) revealed the presence of USUV RNA in the cerebrospinal fluid of a high proportion of people, suggesting that USUV cases in humans may not be a sporadic event [84].

Tick-borne encephalitis virus (TBEV), another orthoflavivirus, has caused epidemics across various Asian and African countries. Cases of TBEV infection have also been reported in several European countries, including Austria, Germany, Switzerland, France, Finland, Norway, Denmark, Slovenia, Romania, Hungary, Poland, Ukraine, and Russia [85].

In addition, recent studies have identified novel orthoflaviviruses such as Aripo virus in Trinidad [86], Menghai orthoflavivirus in China [87], and Hanko virus in northern Europe [88]. Further details on the virological characteristics and genetic variability of notable orthoflaviviruses, such as WNV and DENV, are reviewed in the following sections.

### 2.6. Genome and Replicative Cycle of West Nile Virus and Dengue Virus

**Figure 2:**
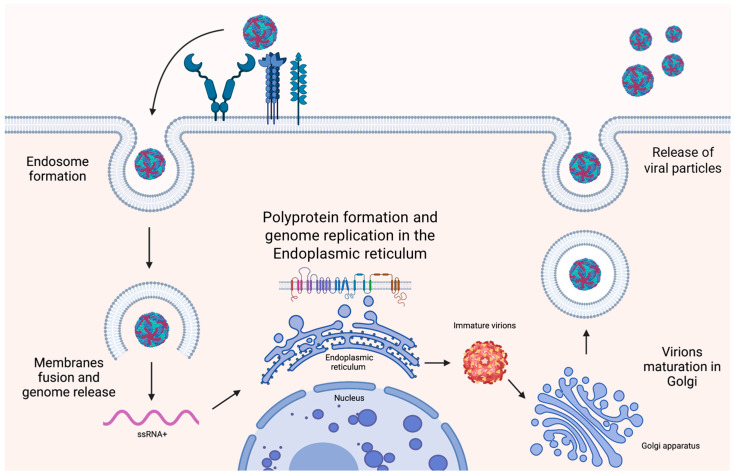
**Orthoflavivirus replicative cycle.** There is an interaction between the viral envelope proteins of West Nile and dengue viruses and various receptors expressed on the cell membrane [e.g., dendritic cell-specific ICAM-3 grabbing non-integrin (DC-SIGN), mannose receptor, and glycosaminoglycans (GAGs)] that mediates endosome formation. Once the endosome is formed, fusion between membranes occurs, resulting in the release of the positive single-stranded (ss)RNA viral genome into the cytosol. The viral genome contains a 5′ internal ribosome entry site (IRES) secondary RNA structure that allows immediate translation into a large polyprotein by the host ribosomes in the endoplasmic reticulum (ER). The polyprotein is then cleaved by proteases into 3 structural proteins and 7 non-structural proteins, including RNA-dependent RNA polymerase, which is responsible for genome replication using a negative (ss)RNA template in the ER. In the ER, virions are assembled and acquire an envelope, but remain immature. The immature virions then move from the ER to the Golgi, where viral maturation takes place. Specifically, in the Golgi, immature virions are processed by the cellular Furin protease, which cleaves the enveloped proteins, resulting in mature, infectious virions. Finally, the viral particles are released by exocytosis. The above figure was created with https://www.biorender.com/ (accessed on 15 July 2024).

The genome of WNV and DENV is a positive (ss)RNA of approximately 11 kilobases (Kb) containing a single open reading frame (ORF) encoding a polyprotein [89] and two non-coding regions (NCR) located at 5′ and 3′ [89]. The polyprotein is cleaved into 10 viral proteins by viral and host proteases [90]. The 5′ terminal NCR encompasses RNA secondary structures, necessary for the translation initiation, the stem loop A (SLA), essential for genome replication, and the internal ribosome entry site (IRES) [91]. After binding to a cellular receptor, viral particles enter the cells by endocytosis and release the nucleocapsid into the cytosol. Once uncoated, the genome is immediately translated into a large polyprotein. Replication of the genome and the formation of new viral particles occur in the endoplasmic reticulum. Lastly, the virions are released from the cell by exocytosis (Figure 2) [92].

### 2.7. Genetic Variability of West Nile Virus

**Figure 3:**
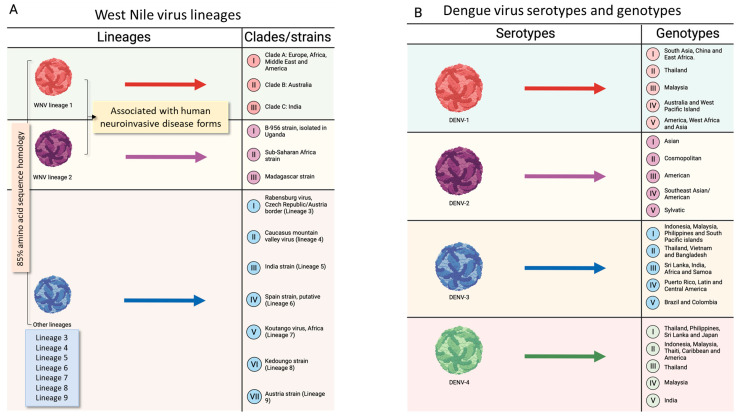
**Taxonomy of West Nile and dengue viruses.** (**A**). The lineages of West Nile virus (WNV) are shown. The first two lineages are found in several regions of the world, while other lineages consist of a few strains that are restricted to specific areas and are not associated with severe disease. (**B**). The serotypes and genotypes of dengue virus (DENV) are shown. DENV is classified into 4 serotypes, each of which can be further subdivided into genotypes (with a maximum of 6% genetic diversity within the same genotype). The above figure was created with https://www.biorender.com/ (accessed on 15 July 2024).

WNV can be distinguished in nine different lineages, but only lineages 1 (WNV-1) and 2 (WNV-2) are of public health interest due to their widespread distribution across continents and the association with human neuroinvasive disease [93]. Specifically, three clades, namely, in alphabetic order, A, B, and C, can be considered for WNV-1 that are distributed in different geographical regions, as shown in Figure 2 [94]. Originally discovered in Uganda in 1937, WNV-2 remained limited to sub-Saharan Africa and Madagascar until the early 2000s (Figure 3) [95]. However, following its outbreak in Hungary and southern Russia [94,96], WNV-2 spread in Europe, causing outbreaks in northern Greece (2010) and in northeastern Italy (2011) [97,98,99]. The migration of birds from African countries to Europe during this period is thought to have caused these outbreaks [99,100]. Other WNV lineages include WNV-3, WNV-4, WNV-5, and WNV-6. The former was isolated on the border between the Czech Republic and Austria from *Cx. pipiens* and *Aedes rossicus* (Dolbeskin, Gorickaja, and Mitrofanova, 1930) and is named Rabensburg virus (RABV) [101]. WNV-4 is a unique virus that was isolated from ticks in the northwestern Caucasus Mountains of Russia in 1998 [102]. WNV-5, referred to as the 1c clade, was isolated in India [103], while WNV-6 is putative and has been found in Spain [104]. WNV-7, known as Koutango virus (WN-KOUTV), has been isolated from ticks and rodents and is confined to Africa [100]. In addition, putative WNV lineages (WNV-8 and WNV-9) have been identified in *Culex perfuscus* (Theobald, 1903) mosquitoes in Kedoungou and *Uranotaenia unguiculata* (Edwards, 1913) mosquitoes in Austria, respectively [105]. Among WNV lineages, there is more than 85% amino acid sequence identity in the polypeptide, and the NS region is highly conserved (Figure 3A).

### 2.8. Genetic Variability of Dengue Virus

The four serotypes of DENV, which are DENV-1, DENV-2, DENV-3, and DENV-4 [106], are subdivided into several genotypes on the basis of the genome analysis of envelope region sequencing. Alongside the four distinct serotypes of DENV, an additional DENV-5 serotype has been detected in a Malaysian man in 2013 [107]. However, the DENV-5 serotype circulates among non-human primates and survives in a sylvatic spreading cycle, suggesting a possible DENV spillover into humans [107].

Concerning DENV-1, it includes five genotypes [108] (I–V): (i) genotype I is found in southeast Asia, China, and the Middle East; (ii) genotype II is widespread in Thailand; (iii) genotype III is characterized by sylvatic strains from Malesia; (iv) genotype IV refers to strains from Pacific Rim countries, Australia, and Western Pacific Islands; (v) genotype V has been isolated from Asia, West Africa, and the Americas. As far as DENV-2 is concerned [109], it is clustered in (i) the Asian genotype, subdivided into Asian 1 (Thailand and Malesia) and Asian 2 (Philippines, Sri Lanka, Vietnam, Taiwan, and China); (ii) the cosmopolitan genotype of Australia, East and West Africa, the Pacific and Indian Ocean islands, the Indian subcontinent, and the Middle East; (iii) the American genotype, isolated in Latin America and the Caribbean; (iv) the southeast Asian/American genotype of Vietnam, Thailand, and several tribes in the Americas; (v) the sylvatic genotype, widespread in West Africa and southeast Asia. Concerning DENV-3, it comprises four genotypes [110] (I–V): (i) genotype I, widespread in the South Pacific islands, Malaysia, Indonesia, and the Philippines; (ii) genotype II strains, found in Thailand, Vietnam, and Bangladesh; (iii) genotype III strains, circulating in Sri Lanka, India, Africa, and Samoa; (iv) genotype IV strains, isolated in Puerto Rico, Latin and Central America, and the 1965 Tahiti strain; (v) genotype V strains, detected in Brazil and Colombia. The last DENV-4 strains can be grouped into five genotypes [111] (I–V): (i) genotype I strains, found in Japan, the Philippines, Thailand, and Sri Lanka; (ii) genotype II strains, found in the Americas, the Caribbean, Indonesia, Malaysia, and Tahiti; (iii) genotype III strains, found in Thailand; (iv) genotype IV strains, found in Malaysia; (v) genotype V, found in India (Figure 3B).

### 2.9. Insight into the Tropism and Pathogenesis of West Nile Virus

**Figure 4:**
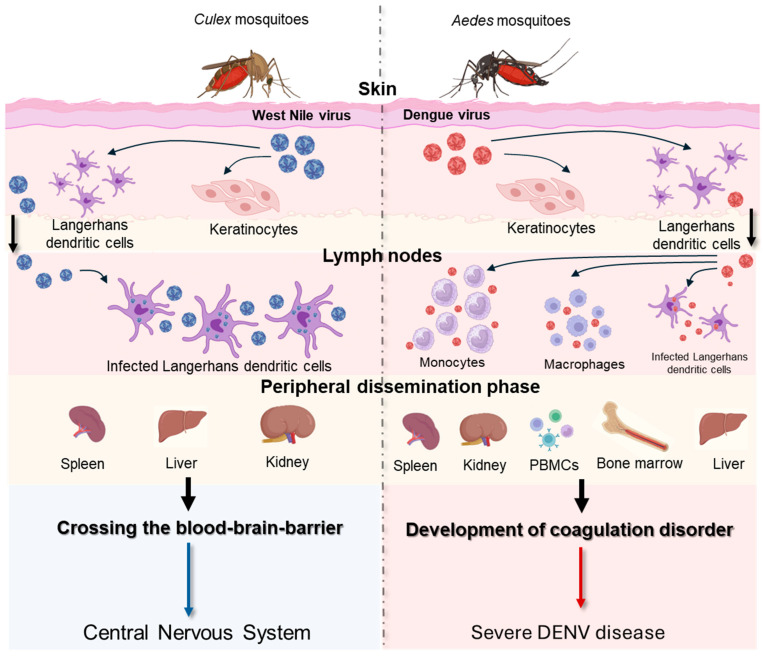
**Pathogenesis of West Nile virus (WNV) and dengue virus (DENV).** When WNV and DENV are inoculated into the skin, both viruses primarily infect Langerhans dendritic cells and keratinocytes [138,139]. Regarding WNV infection, these cells migrate to the lymph nodes and cause primary viremia. Similarly, during DENV infection, Langerhans dendritic cells and keratinocytes migrate in the local draining lymph nodes, where monocytes and macrophages are also recruited and become targets of infection [138,139]. Primary viremia facilitates widespread virus infection across multiple organs. WNV can replicate in various anatomical sites, including the spleen, liver, and kidney, prior to invading the CNS [140]. Concerning DENV, its presence was found in spleen, kidney, peripheral blood, bone marrow, and liver [141]. After peripheral dissemination, WNV crosses the blood–brain barrier (BBB) and causes damage to the CNS. The proposed routes of entry into the CNS of WNV are by passive transport through the endothelium or choroid plex, transport through olfactory neurons, transport through immune-infected cells (Trojan horse), and axonal retrograde transport from infected peripheral neurons [142]. The development of severe DENV disease depends on viral virulence factors and an adverse host immune response that collectively results in abnormal hemostasis and increased vascular permeability [139]. The above figure was created with https://www.biorender.com/ (accessed on 15 July 2024).

**Figure 5:**
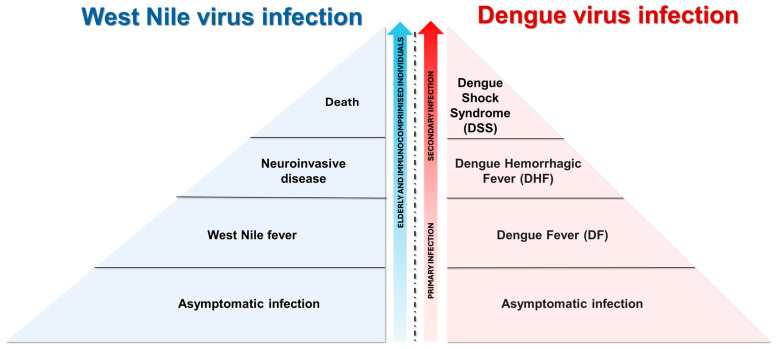
**Clinical manifestation of West Nile virus (WNV) and dengue virus (DENV) infection**. The majority of WNV infections (80%) are asymptomatic, while the remaining of infections (20%) present with flu-like symptoms (i.e., headache, myalgia, fever, maculopapular rash, and gastrointestinal symptoms) [143,144]. Less than 1% of WNV-infected patients develop neuroinvasive diseases such as meningitis and meningoencephalitis and, among them, 10% had fatal outcomes [144,145]. People who are at a greater risk of developing severe WNV infection are the elderly and immune-compromised individuals [146]. Generally, DENV infections are asymptomatic. However, DENV-infected patients may develop symptoms ranging from dengue fever (DF, characterized by mild flu-like syndrome) to dengue hemorrhagic fever (DHF, marked by decreased circulating plasma volume) and dengue shock syndrome (DSS, distinguished by multi-organ failure) [147]. A severe outcome related to DENV infection depends on previous exposure to the virus: heterotypic infections with a different DENV serotype, in comparison to the primary infection, pose the risk for individuals to develop severe illnesses [148]. The above image was created with https://www.biorender.com/ (accessed on 15 July 2024).

Pathogenesis of WNV is shown in Figure 4. Specifically, WNV efficiently establishes its replication on cells that generally express the attachment factors, the adhesion molecule of dendritic cells (DC-SIGN), and the dendritic cell-specific ICAM-3 grabbing nonintegrin-related (DC-SIGNR) [112,113]. One week after virus inoculation, WNV is cleared from the blood and peripheral organs and migrates to the central nervous system (CNS). The mechanism by which WNV crosses the blood–brain barrier (BBB) is not fully understood. It has been shown that higher plasma viremia correlates positively with viral entry into the brain [114]. Several pro-inflammatory cytokines, produced during peripheral immune responses, are involved in the modulation of the BBB. Toll-like receptor 3 (TLR3)-deficient mice, which produce reduced levels of tumor necrosis factor-alpha (TNF-alpha), had less development of neuroinvasive forms compared to wild-type mice [115]. Alongside the compromission of the BBB, WNV can enter the CNS thought alternative mechanisms (Figure 4). In the CNS, neurons, astrocytes, and microglial cells represent target cells for WNV infection, as observed in in vitro studies and in autopsied neural tissues of WNV patients with encephalomyelitis [116,117,118,119,120]. However, differences can be observed between neuronal and glial WNV-infected cells. While rapid WNV replication kinetics and consequent cell death, predominantly apoptosis [121], characterize neuronal cells, slow infection and continuous production of infectious particles are typical of WNV-infected astrocytes [122]. The death of neurons after WNV infection may be also due to neuronophagia, a mechanism by which inflammatory cells, predominantly microglia, phagocytize dying WNV neurons’ infected cells [123]. In general, WNV infection in humans is characterized by a low viral load [<100 plaque-forming unit (PFU)/mL] and a short duration of viremia, occurring one to three days after infection and lasting up to 11 days [124]. However, cases of prolonged infection have been recorded in immunosuppressed patients infected with WNV, where detectable viremia persisted for more than 60 days [125]. Clinical manifestations associated with WNV infection are shown in Figure 5 [126,127]. In addition to the known cycle of WNV between blood-feeding mosquitoes and vertebrate hosts, the potential of this virus to be sexually transmitted has been recognized (Table 2).

### 2.10. Insight into the Tropism and Pathogenesis of Dengue Virus

**Figure 6:**
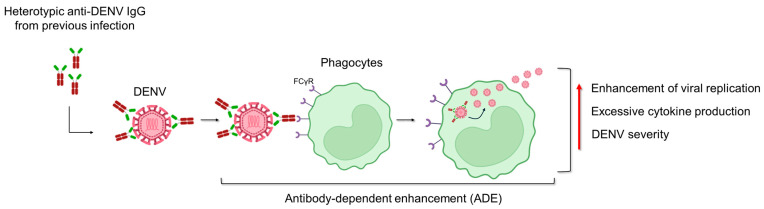
**Antibody-dependent enhancement (ADE).** This phenomenon can be explained by a subset of IgG targeting viral proteins of one dengue virus (DENV) serotype cross-reacting with those of other serotypes and resulting in a poor neutralizing function [149,150,151,152,153]. The antibody–DENV complex binds to the Fcγ receptors (FcγR) on circulating phagocytes (i.e., monocytes), facilitating the infection [154]. This process enhances viral replication and triggers excessive cytokine production. Moreover, ADE initiates an immune cascade that leads to severe dengue disease.

Insights into the pathogenesis of DENV are shown in Figure 4. DENV is able to bind an extensive panel of molecules, in agreement with the wide range of cell types that are susceptible to infection. A variety of host receptor candidates has been proposed, including glycosaminoglycans (GAGs, i.e., heparan sulfate and lectins), the DC-SIGN, mineralocorticoid receptor (MR) of macrophage, and the lipopolysaccharide (LPS) receptor CD14. Moreover, heath shock protein (HSP) 70 and 90 and T-cell/transmembrane, immunoglobulin, and mucin (TIM)-Tyro3, AXL, and MerTK (TAM) are other cellular receptors that DENV uses to promote infection [128,129]. This non-specific targeting allows the virus to disseminate widely throughout the host, leading to the broad range of disease manifestations seen in DENV-infected patients [130]. The pathogenesis of DENV infection encompasses a wide spectrum of dengue disease severity, ranging from mild DF to severe DHF and DSS. DENV viremia is significantly higher in patients with DHF than in those with dengue at one week after symptom onset, suggesting a strong association with disease severity [131]. Severe manifestations of DENV infection are likely to result from a variety of immunopathogenic mechanisms involving viral and host factors. Specifically, hemorrhagic fever is characterized by elevated levels of pro-inflammatory cytokines, referred to as a “cytokine storm”, similar to what has been shown for coronavirus disease 19 (COVID-19) [132,133]. The cytokine storm is characterized by increased plasma levels of TNF-alpha, IL-6, IL-8, IL-10, IL-12, and matrix metalloproteases that are produced by T cells, monocytes, macrophages, and mast cells, which could increase vascular permeability and contribute to hemorrhagic fever [134,135,136,137]. One of the risk factors for the development of severe forms is pre-existing antibodies to DENV. This phenomenon, namely, antibody-dependent enhancement (ADE), is shown in Figure 6. Alongside the primary transmission mode of DENV via mosquito vectors, there are striking recent data concerning the sexual transmissibility of this virus (Table 2).

**Table 2:** Detection of West Nile and dengue virus in the reproductive tract.

Virus	Evidence Supporting WNV and DENV Influence on the Reproductive Tract	References
WNV	Viral RNA has been detected in the semen of a male patient almost 20 days after infection	[155]
	Viral RNA has been detected in post-mortem testicular tissue of a patient with neuroinvasive WNV infection	[156]
	Viral RNA has been detected in the gonadal tissues of crows, ovaries, and testes of deceased parrots	[157,158]
	WNV was suspected to be sexually transmitted one day before the onset of symptoms	[159]
	Fatal WNV infections developed after mice inoculation by the vaginal route	[160]
DENV	DENV sequences were detected in the semen of male patients	[161,162,163,164,165]
	Persistent shedding of DENV RNA was found in the vaginal secretions of a woman more than two weeks after the onset of illness	[166]
	DENV was suspected to be sexually transmitted	[167]

Studies on WNV and DENV localization in the reproductive tract are shown.

## 3. A Six-Year Epidemiological Report on West Nile Virus and Dengue Virus in Europe (2018–2023)

Epidemiological data on the circulation of WNV and DENV in Europe were obtained from the ECDC. Specifically, data for WNV were obtained from the “Annual Epidemiological Reports (AERs) for 2018 and 2019” [168,169], “Epidemiological update: West Nile virus transmission season in Europe for 2020, 2021 and 2022” [170,171,172] and “Weekly updates: 2023 West Nile virus transmission season for 2023” [173]. Data on DENV circulation in Europe were obtained from the “Surveillance Report Dengue annual epidemiological report for 2018, 2019, 2020, 2021 and 2022” [174,175,176,177,178] and from the “Autochthonous vectorial transmission of dengue in Europe Union/European Economic Area (EU/EEA)” [179] section of the ECDC website for 2023. For Italy, the number of DENV infections was collected from the Istituto Superiore di Sanità reports until 2023 (“Bollettini periodici arbovirosi”) [180].

### 3.1. The Number of West Nile Virus Cases in Europe from 2018 to 2023

**Figure 7:**
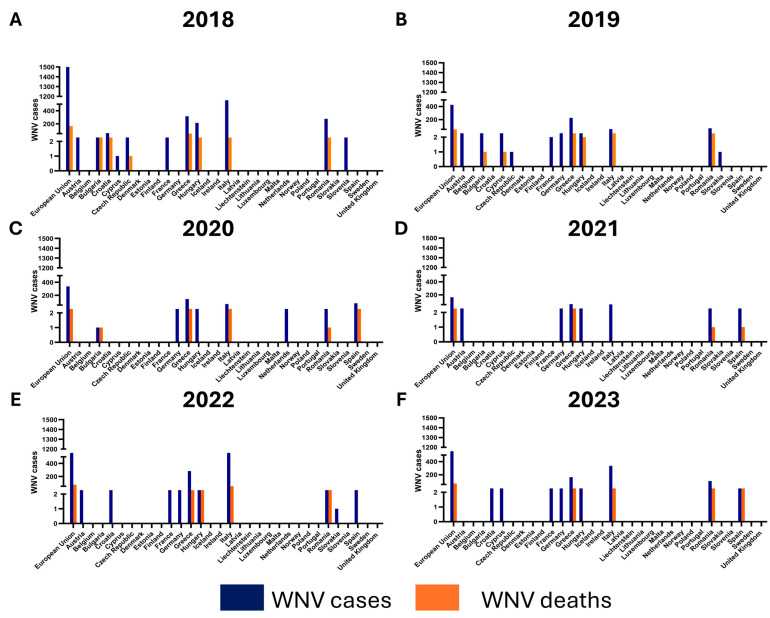
**The number of cases and deaths due to West Nile virus (WNV) infection in European Union countries from 2018 to 2023.** WNV cases and deaths for 2018 (**A**), 2019 (**B**), 2020 (**C**), 2021 (**D**), 2022 (**E**) and 2023 (**F**) are shown. Data were collected from the European Centre for Disease Prevention and Control (ECDC) reports updated on 31 December 2023 (“West Nile virus infection—Annual epidemiological report for 2018”, 2019; “West Nile virus infection—Annual epidemiological report for 2019”, 2020; “Epidemiological update: West Nile virus transmission season in Europe, 2020”, 2021; “Epidemiological update: West Nile virus transmission season in Europe, 2021”, 2022; “Epidemiological update: West Nile virus transmission season in Europe, 2022”, 2023; “Weekly updates: 2023 West Nile virus transmission season, 2023”).

All WNV cases in the European Union (EU) are shown in Figure 7. Considering all EU cases from 2018 to 2023, the number of WNV cases decreased from 2018 to 2021, while a new peak of cases was observed in 2022 [168,169,170,171,172]. Between 2018 and 2023, 406 deaths were reported among cases of WNV infection, with a peak registered in 2018, which corresponds to the highest number of WNV human cases reported in that year (Figure 7) [168,169,170,171,172,173]. In European countries where WNV is endemic, COVID-19 restrictions had little or no effect. This is consistent with what has been observed in avian surveillance, where it coincides with an epidemic in these animals [181]. Over the years, most of the cases have been observed in Mediterranean EU countries, including Italy and Greece, where summer temperatures are usually high, allowing mosquitoes to spread. Interestingly, WNV was not detected in both birds and humans in Germany before 2018, while the first WNV cases were observed in birds but not in humans in 2018 [182]. In addition, two outbreaks of WNV in horses were observed in September and October 2018 [183]. Insecticide resistance in *Cx. pipiens* mosquitoes has also been reported in Europe, posing a significant threat to the spread of mosquito-borne disease vectors. Recent data examining the evolution and spread of WNV in Europe suggest that, in addition to ecological conditions favoring the presence of birds and mosquitoes, the intensity of agricultural activity may be an important driver of WNV emergence and spread [184].

### 3.2. The Number of Dengue Virus Cases in Europe from 2018 to 2023: The Emergence of Dengue Virus Autochthonous Cases

**Figure 8:**
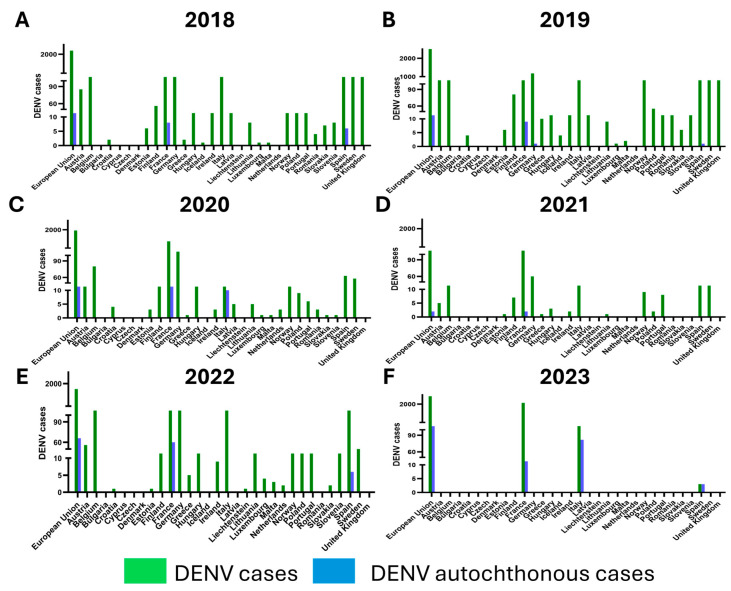
**Total and autochthonous dengue virus (DENV) cases in European Union (EU) countries**. Total and autochthonous DENV cases for 2018 (**A**), 2019 (**B**), 2020 (**C**), 2021 (**D**), 2022 (**E**) and 2023 (**F**) are shown. All DENV cases, including autochthonous and imported cases, found in Europe between 2018 and 2022 were collected from the European Centre for Disease Prevention and Control (ECDC) reports (“Surveillance Report Dengue annual epidemiological report for 2018”, 2019; “Surveillance Report Dengue annual epidemiological report for 2019”, 2021; “Surveillance Report Dengue annual epidemiological report for 2020”, 2022; “Surveillance Report Dengue annual epidemiological report for 2021”, 2023; “Surveillance Report Dengue annual epidemiological report for 2022”, 2024). Annual ECDC reports on the total number of cases in Europe, the sum of travelers returning from dengue-endemic areas, and autochthonous cases for 2023 are not yet available. The only available data on imported cases of DENV infections for 2023 are for France and Italy, respectively, from the “Santè publique France” and “Istituto Superiore di Sanità” websites [197,199]. In parallel, data on autochthonous DENV infections in Europe in 2023 are summarized on the ECDC website in the section “Autochthonous vectorial transmission of dengue in EU/EEA” [179].

DENV cases in Europe are mainly imported cases, due to infected people who have traveled abroad (Figure 8). Most of the DENV cases in the EU were observed in 2019; in parallel, DENV epidemics were reported in many countries around the world in the same year, including Bangladesh [185], Nepal [186], and Honduras [187]. Besides the recent impact of the spread of SARS-CoV-2 on the circulation of DENV, autochthonous cases have been reported in Europe over the past decade, probably due to the establishment of *Ae. albopictus* on the continent. The first autochthonous cases of DENV infection in the EU were described in 2010, in Croatia and France (15 and 2 cases, respectively) [188,189]. Several human cases of autochthonous DENV infection were then reported in France until 2015 (1 case in 2013, 4 cases in 2014, and 8 cases in 2015) [190,191]. In 2018, 6 autochthonous cases of DENV were reported in France [192], and it was also the first year in which autochthonous DENV cases were reported in Spain [193]; in particular, 3 cases were observed in August (Autonomous Communities of Murcia and Andalusia) (none of the DENV-infected individuals had traveled to endemic areas in the previous 2 weeks before symptom onset) and the other 3 cases in October, geographically close to the first cases (Autonomous Community of Murcia). Sequencing showed that DENV-1 caused the outbreak in Spain. However, no *Ae. albopictus* mosquitoes were positive for DENV-RNA detection. One novel DENV human infection was reported in Germany, but this was linked to an infection in a virology laboratory [175]. In 2022, 66 autochthonous vector transmissions of DENV were observed in mainland EU/EEA, including 60 and 6 cases in France and Spain, respectively [178]. In 2023, a slight decrease of DENV in non-travel-associated infections was recorded, with 45 cases in France and 3 cases in Spain [179].

Italy reported its first autochthonous cases in 2020 (see further section below). In 2021, there was a general decrease in human DENV infections, probably due to COVID-19 travel restrictions, but 2 new autochthonous cases were identified in France [194]. In contrast to 2022, when no autochthonous cases were reported in Italy, a new peak of 82 non-travel-associated DENV infections was recorded in 2023 [195,196,197,198].

### 3.3. West Nile Virus Circulation in Italy

**Figure 9:**
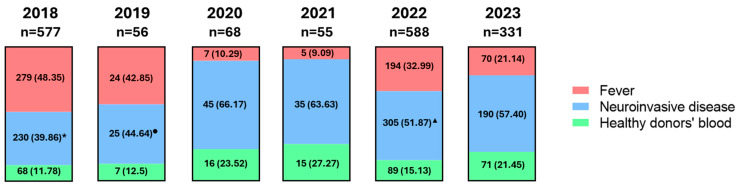
West Nile virus (WNV) cases in Italy from 2018 to 2023 according to clinical manifestations (fever, neuroinvasive disease, and cases in blood donors). All data were collected from reports of the “Istituto Superiore di Sanità” (“Sorveglianza integrata del West Nile e Usutu virus 2018”, 2018; “Sorveglianza integrata del West Nile e Usutu virus 2019”, 2019; “Sorveglianza integrata del West Nile e Usutu virus 2020”, 2020; “Sorveglianza integrata del West Nile e Usutu virus 2021”, 2021; “Sorveglianza integrata del West Nile e Usutu virus 2022”, 2022; “Sorveglianza integrata del West Nile e Usutu virus 2023”, 2023). * There were 42 deaths reported in 2018; ^●^ There were 5 deaths reported in 2019; ^▲^ There were 37 deaths reported in 2022.

Human WNV infections from 2018 to 2023 are shown in Figure 9. Analyses of *Cx. pipiens* mosquitoes, the vector for WNV in Italy, collected from 2018 to 2020 showed the circulation of WNV-2 in Italy during these years. The first circulations in Italy of both WNV-1 and WNV-2 were detected in human WNV infections in 2021. In particular, WNV-1 human infections were reported in 2021 in the provinces of Padua (n = 2) and Vicenza (n = 1) [200]. In the same year, WNV-2 was detected in a patient with neuroinvasive disease and in a blood donor [200]. In 2022, the presence of WNV-1 was confirmed in 2 human cases (i.e., a blood donor and a patient with encephalitis) in the province of Padua. With regard to WNV-2, in 2022, one WNV nucleic acid test (NAT)-positive blood donor was identified in the province of Venice. Regarding the circulation of WNV lineages in 2023, veterinary surveillance conducted on horses, mosquitoes, and birds confirmed the presence of WNV-2 in northern Italy (Emilia Romagna, Friuli Venezia Giulia, Liguria, Marche, Veneto, Lombardy, and Piedmont), as well as in Apulia, Sardinia, and Sicily [201]. At the same time, the circulation of WNV-1 was restricted to Emilia-Romagna, Sicily, Veneto, and Campania [201].

### 3.4. Dengue Virus Circulation in Italy

**Figure 10:**
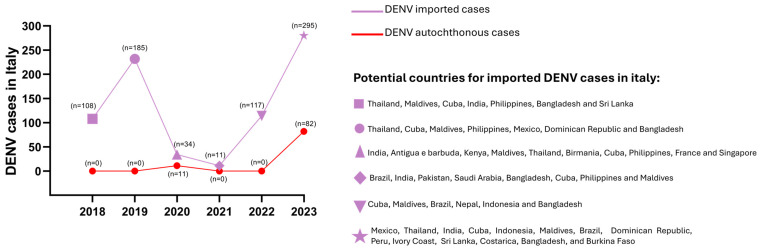
**The number of dengue virus (DENV) cases, including autochthonous cases, in Italy from 2018 to 2023.** Dengue endemic countries were reported for imported cases. All data were collected from reports of the Istituto Superiore di Sanità (“Arbovirosi in Italia. Dengue. Anno 2018”, 2018; “Arbovirosi in Italia. Dengue. Anno 2019”, 2019; “Arbovirosi in Italia. Dengue. Anno 2020”, 2020; “Arbovirosi in Italia. Dengue. Anno 2021”, 2021; “Arbovirosi in Italia. Dengue. Anno 2022”, 2022; “Arbovirosi in Italia. Dengue. Anno 2023”, 2023).

Data on DENV infections in Italy from 2018 to 2023 are shown in Figure 10. DENV infections in Italy are mainly imported as the mosquito vector of DENV (*Ae. aegypti*) is not present in the country. Notably, the first autochthonous cases in Italy, likely due to the presence of *Ae. albopictus* in Italy, were identified in August 2020. In particular, a family cluster of DENV infections was reported in the province of Vicenza (northeastern Italy), starting from a traveler returning from Indonesia with DENV symptomatic infection. One month after the onset of symptoms, six additional household members became infected with DENV-1, as did other individuals living in the vicinity of the primary case [202].

During the COVID-19 pandemic, there was a reduction of imported DENV cases from 185 in 2019 to 34 (−80.6% compared to the previous year) in 2020 and 11 (−75.6% compared to the previous year) in 2021 [175,177]. By contrast, an increased rate of DENV infections was described in 2022, with a total of 117 new DENV cases, as SARS-CoV-2 restrictions were almost completely removed. Imported DENV cases in Italy derived mainly from Thailand, the Maldives, and Cuba, where DENV is considered endemic [203,204,205]. Among EU countries, France had the highest number of autochthonous DENV cases, with a peak of DENV infections (65 confirmed cases) in 2022 [190]. It was considered the most likely country responsible for the imported DENV cases described in 2022 from Italy [179].

Data on DENV infections in Italy in 2023 reported 295 reported cases in travelers returning from dengue-endemic regions (mainly Mexico, Thailand, India, Cuba, and Indonesia); 1 of these DENV cases resulted in death [195,197]. In parallel, 82 new autochthonous cases were registered in the province of Lodi, Lombardy (41 cases of DENV-1 infection) [204,206], in the province of Latina (2 cases), in Rome (38 cases), and in Anzio, Lazio region (1 case) [12,198].

## 4. Conclusions

The latitudinal range of mosquitoes and other arthropods is expected to expand, allowing these disease-carrying insects to spread from the tropics to temperate regions where they were previously absent. With rising temperatures and the effects of global warming, vector capacity has increased and will continue to increase. As a result, there will be an increase in (i) the number of daily bites; (ii) the likelihood of disease transmission from the vector to humans; (iii) the likelihood of the vector becoming infected; (iv) the long incubation period of the pathogen; (v) the time between the moment the pathogen is ingested and the moment a mosquito can transmit the disease; (vi) the mortality and reproduction rate of the vector. Peaks in WNV infections were reported in Italy in 2018 and 2022. These events were partly attributed to rising temperatures followed by rainfall, which facilitates the spread of *Cx. pipiens*. As for DENV infection, it remains the most commonly identified cause of fever in sick international travelers, as confirmed by numerous cases of DENV in Europe. However, the recent outbreak of autochthonous cases of dengue virus (DENV) in Europe has become an urgent concern. A notable increase in autochthonous cases occurred in Italy in 2023, highlighting the urgent need to strengthen DENV surveillance in the country and the potential for tropical disease outbreaks in non-endemic regions where competent vectors may be present.
